# Genetic diversity and population structure of *Cynara cardunculus L*. in southern Portugal

**DOI:** 10.1371/journal.pone.0252792

**Published:** 2021-06-09

**Authors:** Maria Miguel Castro, Daniela Rosa, Ana M. Ferro, Ana Faustino, Ana Paulino, Teresa Brás, Eliana Machado, Carla Pinto Cruz, Anabela D. F. Belo, Paula Nozes, João Portugal, Sofia Ramôa, Diogo Mendonça, Fernanda Simões, Maria F. Duarte, Liliana Marum

**Affiliations:** 1 Centro de Biotecnologia Agrícola e Agro-Alimentar do Alentejo (CEBAL)/Instituto Politécnico de Beja (IPBeja), Beja, Portugal; 2 MED – Mediterranean Institute for Agriculture, Environment and Development – CEBAL, Beja, Portugal; 3 Centre for Ecology, Faculdade de Ciência, Evolution and Environmental Changes (cE3c), Universidade de Lisboa, Lisboa, Portugal; 4 LAQV/ REQUIMTE, FCT, Universidade Nova de Lisboa, Caparica, Portugal; 5 MED-Instituto Mediterrâneo para a Agricultura, Ambiente e Desenvolvimento, Universidade de Évora, Ap 94, Évora, Portugal; 6 Ambiente e Desenvolvimento & Departamento de Biologia, MED - Instituto Mediterrâneo para a Agricultura, Escola de Ciências e Tecnologia, Universidade de Évora, Ap 94, Évora, Portugal; 7 Departamento de Biociências/Instituto Politécnico de Beja (IPBeja), Beja, Portugal; 8 VALORIZA – Centro de Investigação para a Valorização dos Recursos Endógenos, Instituto Politécnico de Portalegre, Portalegre, Portugal; 9 Instituto Nacional de Investigação Agrária e Veterinária I.P. (INIAV IP), Unidade Estratégica de Biotecnologia e Recursos Genéticos, I.P., Oeiras, Portugal; Brigham Young University, UNITED STATES

## Abstract

*Cynara cardunculus* L. is a cardoon species native to the Mediterranean region, which is composed of three botanical taxa, each having distinct biological characteristics. The aim of this study was to examine wild populations of *C*. *cardunculus* established in Portugal, in order to determine their genetic diversity, geographic distribution, and population structure. Based on SSR markers, 121 individuals of *C*. *cardunculus* from 17 wild populations of the Portuguese Alentejo region were identified and analysed. Ten SSRs were found to be efficient markers in the genetic diversity analysis. The total number of alleles ranged from 9 to 17 per locus. The expected and observed means in heterozygosity, by population analysed, were 0.591 and 0.577, respectively. The wild population exhibited a high level of genetic diversity at the species level. The highest proportion of genetic variation was identified within a geographic group, while variation was lower among groups. Geographic areas having highest genetic diversity were identified in Alvito, Herdade da Abóboda, Herdade da Revilheira and Herdade de São Romão populations. Moreover, significant genetic differentiation existed between wild populations from North-Alentejo geographic locations (Arraiolos, Évora, Monte da Chaminé) and Centro Hortofrutícola, compared with other populations. This study reports genetic diversity among a representative number of wild populations and genotypes of *C*. *cardunculus* from Portugal. These results will provide valuable information towards future management of *C*. *cardunculus* germplasm.

## Introduction

*Cynara cardunculus* L. is a perennial species native to the Mediterranean area and is well adapted to hot and dry climates. It belongs to the Asteraceae family, and it comprises three botanical varieties: *C*. *cardunculus* var. *scolymus* (L.) Fiori (globe artichoke), *C*. *cardunculus* var. *altilis* (DC). (cultivated cardoon) and *C*. *cardunculus* var. *sylvestris* (Lamk) Fiori (wild cardoon) [1] *C*. *cardunculus* is diploid (2n = 2x = 34) and allogamous species. Crosses between members of the three varieties are highly variable, conferring a wide degree of genetic and phenotypic diversity [1–5].

The globe artichoke has been widely used for human consumption, in southern Europe, mainly Portugal, Spain and Italy, while wild cardoon can also serve as a rennet for production of cheese, resulting from aspartic proteinases activity from its flower heads [6–8]. Additionally, *C*. *cardunculus* has been worldwide cultivated and investigated as a potential source of solid biofuel/lignocellulosic biomass, seed oil, biodiesel, paper pulp, green forage and pharmacologically active compounds [9–11]. As a source of several nutraceutical and pharmaceutical compounds, such as phenylpropanoids and sesquiterpenes [12–20], due their biological activity, *C*. *cardunculus* has been well studied regarding its biological potential. According to studies that characterized chemically the different parts of *C*. *cardunculus* plants [leaves, stalks and capitula (receptacle and bracts, and florets) [21, 22]], cardoon is a rich source of different valuable compounds. Among them, we highlight the sesquiterpene lactones (SL) present in *C*. *cardunculus* leaves (94.5 g/kg dry weight), with cynaropicrin as the major SL presented (87.5 g/kg dry weight) [21, 23]. Cynaropicrin biological potential is well described, as well as its use in the food industry [24, 25].

In order to develop a breeding strategy to enhance content of bioactive compounds, having pharmaceutical and/or nutraceutical applications, it is advantageous to screen *C*. *cardunculus* genetic diversity. Genetic markers, such as microsatellites (SSRs, simple sequence repeats), are informative molecular markers and are useful in breeding programs for marker-assisted selection. The technical ability to characterize germplasm [26] and detect the basis of complex genetic traits of *C*. *cardunculus* has vastly improved the ability to construct genetic linkage maps [27]. Since the introduction of the first linkage map for globe artichoke [5], studies have identified and located major loci controlling key agronomic traits of *C*. *cardunculus*, based on different classes of molecular markers [28]. More recently, the sequences of the globe artichoke nuclear genome [29] and of the chloroplast genome of *C*. *cardunculus* taxa [30, 31] have been described.

While there have been some studies of genetic diversity and genetic relationships between cardoon from certain European countries and Tunisia [32–35], there is little information available on the genetic background of wild Portuguese populations of *C*. *cardunculus*. In order to fill this gap, our study aimed to gain knowledge on *C*. *cardunculus* genetic diversity and population structure in Portuguese cardoon by characterizing genotypes from multiple geographic locations using SSR markers.

## Materials and methods

### Plant material

**Fig 1:**
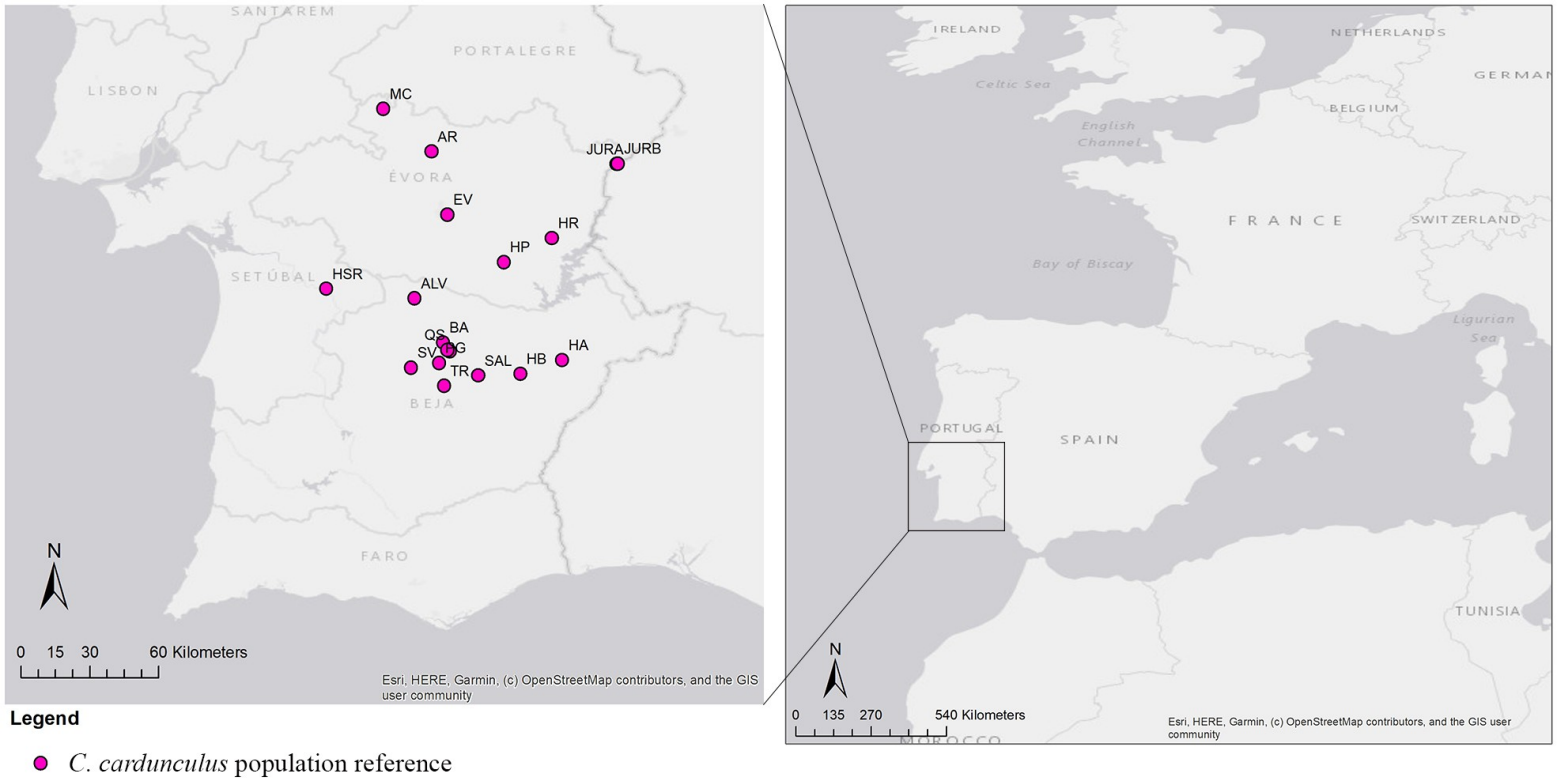
Maps indicating geographic locations and distribution of the wild populations of *Cynara cardunculus*, sampled in Portugal.

Samples of *Cynara cardunculus* L. (121 individuals) were collected from populations found in the wild in 17 geographic locations, distributed across the Alentejo region in southern Portugal (see Table 1; Fig 1), during June-July 2016 and 2017. In result of the not so clear taxonomy of intraspecific taxa of *C*. *cardunculus* in the wild [36], we chose to refer to the cardoon plants of our study just as *C*. *cardunculus*.

**Table 1:** Abbreviated reference names, population origin and geographic locations of *Cynara cardunculus* samples.

Reference name (Abbr.)		Field-plot name	Field-plot Location	No. genotypes sampled	Biological status	Geographical coordinates	
						Latitude	Longitude
**CH**		Centro Hortofrutícola	Beja, PT	7	Indeterminate	38°01`57.1”	‒007°52`29.6”
**BA**		Base Aérea	Beja, PT	7	Wild	38°03`55.21”	‒007°54`01.53”
**QS**		Quinta da Saúde	Beja, PT	7	Wild	38°02`12.6”	‒007°53`02.0”
**PG**		Penedo Gordo	Penedo Gordo, Beja, PT	7	Wild	37°59`12.4”	‒007°55`00.1”
**SAL**		Salvada	Salvada, Beja, PT	7	Wild	37°56`16.3”	‒007°45`47.0”
**SV**		Santa Vitória	Santa Vitória, Beja, PT	7	Wild	37°58`09.5”	‒008°01`35.1”
**HB**		Herdade dos Barretos	Serpa, Beja, PT	7	Wild	37°56`39.9”	‒007°35`52.2”
**HA**		Herdade da Abóbada	Vila Nova de São Bento, Beja, PT	9	Wild	37°59`51.5”	‒007°26`00.8”
**ALV**		Alvito	Alvito, Beja, PT	7	Wild	38°14`08.1”	‒008°00`44.1”
**MC**		Monte da Chaminé	Mora, Évora, PT	7	Wild	38°57`56.2”	‒008°08`06.1”
**HP**		Herdade do Peral	Monte do Trigo, Évora, PT	7	Wild	38°22`32.8”	‒007°39`45.9”
**HR**		Herdade da Revilheira	Santo António do Baldio, Évora, PT	7	Indeterminate	38°28`02.6”	‒007°28`27.4”
**HSR**		Herdade de São Romão	São Romão, Sétubal, PT	7	Wild	38°16`19.9”	‒008°21`33.7”
**JUR**	A	Juromenha A	Juromenha, Évora, PT	4	Wild	38°45`08.6”	‒007°13`17.3”
	B	Juromenha B		3	Wild	38°45`18.1”	‒007°12`59.9”
**TR**		Trindade sem picos	Trindade, Beja, PT	7	Wild	37°53`56.2”	‒007°53`48.3”
**AR**		Arraiolos	Arraiolos, Évora, PT	7	Wild	38°44`02.12”	‒007°56`48.32”
**EV**		Évora	Évora, PT	7	Wild	38°33`29.58”	‒007°53`06.29”
**TOTAL**				**121**			

From each location, seven to nine different genotypes were selected for genetic analysis. In total, samples (leaves) of *C*. *cardunculus* included 121 genotypes from 17 populations (Table 1; Fig 1), leaves were air-dried and ground to a powder for DNA isolation.

### DNA isolation

Total genomic DNA was extracted from samples using the DNeasy Plant Mini Kit (Qiagen, Germany), following manufacturer protocols. Prior to PCR amplification, DNA concentration and purity were determined spectrophotometrically (NanoVue plus, GE Healthcare Life Sciences, USA), while DNA integrity was assessed electrophoretically on 1% agarose gels, stained with GreenSafe Premium (NZYTech, Portugal) using a GelDoc XR System (BioRad, USA) for image capture and analysis.

### Microsatellite analysis

Twenty-three pairs of primers for microsatellite analysis were retrieved from the available literature for artichoke [37–39] (S1 Table). Pairs of primers were chosen according to linkage-group positions and to level of potential polymorphism.

PCR amplifications were performed using a total volume of 20 μL containing 0.1–0.5 ng of genomic DNA, 0.5 μM of forward and reverse primers and 1x Dream Taq PCR Mastermix (Thermo Scientific, USA). Amplification was performed using the following conditions: initially 95 °C—10 min; then 40 cycles at 95 °C– 45 s; optimal annealing temperatures respective to primers—60 s (Table 2); 72 °C– 45 s; and a final elongation at 72 °C—10 min. Controls lacking a DNA template were included in each PCR reaction for each primer pair. Only ten of twenty three pairs of primers listed in S1 Table with amplification and specific PCR products were used for the following fragment analysis.

**Table 2:** Genetic parameters based on 10 Simple Sequence Repeat (SSR) loci and 121 *Cynara cardunculus* individuals.

SSR Loci Name	Ta	N	Ho	He	I	PIC
	(°C)					
**CELMS-05**	53	15	0.442	0.528	0.985	0.702
**CELMS-61**	53	13	0.735	0.675	1.290	0.805
**CyEM-138**	60	16	0.674	0.644	1.261	0.823
**CELMS-58**	60	17	0.708	0.693	1.369	0.867
**CyEM-183**	55	9	0.554	0.499	0.804	0.487
**CyEM-229**	55	15	0.584	0.584	1.091	0.752
**CELMS-03**	51	16	0.634	0.591	1.133	0.813
**CELMS-11**	51	14	0.642	0.624	1.219	0.850
**CELMS-14**	51	13	0.664	0.648	1.292	0.817
**CELMS-17**	51	10	0.134	0.423	0.695	0.497
**Total**		138				
**Mean**		13.8	0.577	0.591	1.114	0.741

Note: Ta-annealing temperature; N-number of alleles; Ho-observed heterozygosity; He-expected heterozygosity; I-Shannon’s Information Index; PIC-Polymorphic information content.

Amplicons of each primer pair were sequenced using the ABI 3730xl platform, to confirm the specificity of PCR products. Thereafter, multiplex PCRs were carried out using NYZProof DNA polymerase 2x Colourless Master Mix (NYZTech, Portugal) according to manufacturer’s instructions, using the annealing temperatures listed in Table 2. Each forward primer was fluorescent-labelled with 6-FAM or HEX dyes (STAB VIDA, Portugal), and two loci were amplified in the same reaction, according to the combinations described in S1 Table. PCR was carried out as follows: 95 °C—3 min; followed by 40 cycles at 94 °C—30 s, respective annealing temperature (Table 2) - 45 s; 72 °C—45 s; and final extension at 72 °C—10 min.

All PCR amplifications were performed using a MyCycler (BioRad, USA) thermocycler. For fragment analysis, PCR products were separated by capillary electrophoresis on an ABI 3130 Genetic Analyser (Applied Biosystems, Foster City, CA, USA) and peaks identified using internal size GeneScan^™^ 500 LIZ^®^ Size Standard (Applied Biosystems) (S1 Fig). DNA fragment lengths were determined using GeneMapper software (Applied Biosystems, USA).

### Data analysis

Genetic diversity parameters, such as total number of alleles (N) and polymorphic information content (PIC), were calculated using PowerMarker v.3.25 software [40]. Mean number of alleles (Na), expected heterozygosity (He), observed heterozygosity (Ho), Shannon’s diversity index (I) and Fixation Index *F* (Inbreeding Coefficient) and Wright’s F_ST_ used to estimate genetic diversity and population differentiation, were generated by GenALEx v 6.5 software [41]. Analysis of molecular variance (AMOVA) was also performed using GenAlEx v6.5, to evaluate genetic variation among and within populations. Genetic distance was estimated according to Nei parameter [42]. MICROCHECKER [43] was used to test for the possibility of scoring errors, allelic dropout, and null alleles. Principal coordinate analyses (PCoA) [44] were performed, using GenAlEx v6.5 to identify genetic variation patterns among *C*. *cardunculus* genotypes. Genetic dissimilarity matrices and neighbour-joining (NJ) cluster analyses were used to construct genetic affiliation trees using Darwin v.6 software [45].

Population structure was performed using the Bayesian model-based clustering approach, using software STRUCTURE v2.3.4 software [46], to elucidate relationships among populations. Initially, geographic populations were assigned to 17 groups (Table 2). Number of populations (K) was estimated by performing five independent runs for each K (from 1 to 10), using 100 000 MCMC steps and 50 000 burn-in periods, assuming the following parameters: admixture model and correlated allele frequencies model. The optimum number of populations (K) was processed and identified by STRUTURE HARVESTER web v 0.6.94, July2014 by comparing log probabilities of data for each value of K [47, 48]. The clustering pattern was visualised using the Structure Plot V2 [49].

## Results

Having a thorough comprehension of the genetic diversity and population structure of wild *C*. *cardunculus*, in Portugal, is an important step towards using available genetic resources, to develop cultivars useful to agriculture and industry. Until now, available knowledge regarding cardoon germplasm in the Iberian Peninsula was limited. The results of our study significantly enhance that knowledge.

For our study, SSR markers were used to characterize genetic diversity of 121 genotypes of *C*. *cardunculus* collected across the Alentejo region (southern Portugal), from 17 Portuguese populations. For this characterization we analyzed several parameters including, number of alleles (N), polymorphic information content (PIC), number of different alleles (Na), number of effective alleles (Ne), Shannon’s Information Index (I), observed (Ho) and expected (He) heterozygosity, and Fixation Index (F).

### Diversity parameters for *Cynara cardunculus* individuals

For genetic characterization, initially 23 SSRs were used for microsatellite screening of the cardoon collection. Of these, only 10 showed reproducible and specific PCR amplification, as confirmed by sequencing (S2 Table). A total of 138 alleles were generated from all 121 *C*. *cardunculus* individuals under study. The number of alleles *per* locus showed a range from nine (CyEM-183) to 17 (CELMS-58), with an average of 13.8 (Table 2; S3 Table).

He values for loci had a range from 0.423 (CELMS-17) to 0.693 (CELMS-58), with an average of 0.591, while Ho had a range between 0.134 (CELMS-17) and 0.735 (CELMS-61), with an average of 0.577 (Table 2).

Average PIC was 0.741, varying from 0.487 (CyEM-183) to 0.867 (CELMS-58). The majority of optimized markers were highly informative (PIC≥0.70). The Shannon information index varied from 0.695 (CELMS-17) to 1.369 (CLEMS-58), with an average of 1.114 (Table 2).

These results demonstrated that the employed SSR markers were effective in providing valid estimates of genetic diversity of the cardoon population, as represented by the average of genetic diversity indices (PIC = 0.741, He = 0.591, I = 1.114).

### Genetic diversity of *Cynara cardunculus* populations

Genetic diversity analysis were performed for all *C*. *cardunculus* populations (Table 3). Among populations, the number of different alleles Na varied from 2.7 (TR) to 5.5 (HA), with an average of 4.100. The number of effective alleles, Ne, had a range from 1.961 (AR) to 3.713 (HA) and an average of 2.912. Ho values for each geographic population ranged from 0.443 (EV) to 0.733 (HA), with an average of 0.577. Whereas, expected heterozygosity had a variation from 0.419 (AR) to 0.700 (ALV) with an average of 0.591. The Shannon information index had a range from 0.737 (AR) to 1.388 (HA) (Table 3).

**Table 3:** Genetic diversity analyses of *Cynara cardunculus* from 17 populations.

Geographic population	Na	Ne	I	Ho	He	F
**CH**	3.000	2.295	0.867	0.657	0.506	-0.305
**BA**	3.500	2.518	0.983	0.514	0.555	0.057
**QS**	4.700	3.366	1.266	0.614	0.639	0.076
**PG**	4.200	2.808	1.161	0.629	0.630	-0.008
**SAL**	4.300	2.971	1.146	0.571	0.592	0.094
**SV**	4.300	3.038	1.180	0.600	0.621	0.033
**HB**	4.300	2.998	1.124	0.529	0.579	0.088
**HA**	5.500	3.713	1.388	0.733	0.689	-0.031
**ALV**	4.500	3.572	1.332	0.714	0.700	-0.023
**MC**	3.600	2.542	0.988	0.514	0.542	0.074
**HP**	4.400	3.33	1.223	0.490	0.641	0.257
**HR**	5.300	3.499	1.358	0.586	0.668	0.171
**HSR**	5.300	3.411	1.376	0.600	0.684	0.150
**JURA/B**	4.000	3.017	1.201	0.586	0.654	0.097
**TR**	2.700	2.037	0.761	0.557	0.458	-0.227
**AR**	3.000	1.961	0.737	0.471	0.419	-0.143
**EV**	3.100	2.434	0.844	0.443	0.465	0.039
**Mean**	4.100	2.912	1.114	0.577	0.591	0.026

Note: Na—Number of different alleles; Ne- Number of effective alleles; I-Shannon’s Information Index; Ho-observed Heterozygosity; He-Expected Heterozygosity; F-Fixation Index.

Fixation indices revealed the existence of at least two distinct groups showing values round zero and/or negative. The BA, QS, SAL, SV, HB, MC, EV, JRA/B, HR, HP and HSR populations were more homozygous than expected (F positive). CH, PG, ALV, TR and AR populations showed negative F values, indicating significantly higher heterozygosity than expected, possibly resulting from negative assortative mating or selection for heterozygotes. MICROCHECKER analysis did not detect evidence for scoring errors due to stuttering, neither for allele dropout, nor for a high frequency of null alleles in any of the tested loci, although still not perturbing Hardy-Weinberg equilibrium of the natural populations.

### Genetic differentiation within and among geographic populations

Analysis of molecular variance (AMOVA) for genetic differentiation among and within populations of *C*. *cardunculus* showed only an occurrence of 14% of genetic variation occurred among populations (Table 4). Contrastingly, 86% of remaining variability in genetic variation was represented within the population.

**Table 4:** Analysis of Molecular Variance (AMOVA) within/among *Cynara cardunculus* populations.

Source	df	SS	MS	Est. Var.	%
**Among Populations**	16	88644.178	5540.261	273.621	14%
**Within Populations**	225	370406.798	1646.252	1646.252	86%
**Total**	241	459050.975		1919.873	100%

Note: df, degrees of freedom; SS, sum of squared; MS, mean squared; Est.Var., Estimated variance; %, percentage of AMOVA values.

**Fig 2:**
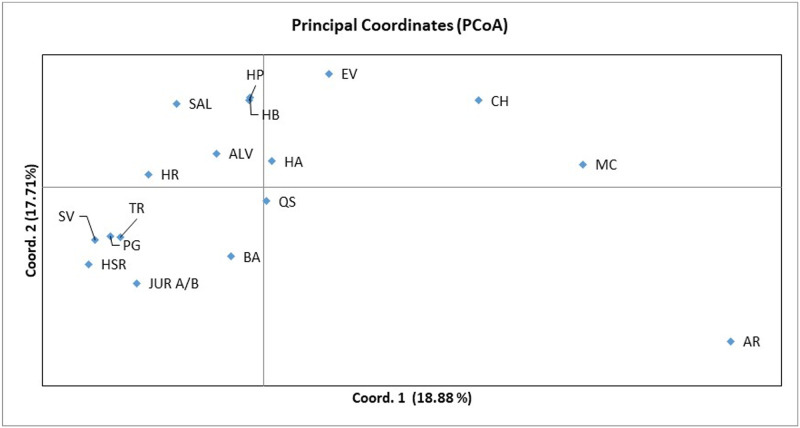
Principal coordinate analysis of Nei’s genetic distance for the 17 geographic populations of *Cynara cardunculus*.

The results of principal coordinate analysis (PCoA), based on Nei’s genetic distance, are presented in Fig 2. The first two coordinates of the analysis account for 36.6% of the total variation. The first coordinate explains 18.88% of the variation and indicates mainly, the degree of separation of AR, MC, CH, EV from the remaining populations. The second coordinate explains an additional 17.71% of the variation (Fig 2 and S4 Table).

Pairwise *F*_ST_ values, a measure of genetic differentiation among populations, showed the most differentiated wild populations in *C*. *cardunculus* were EV and AR (*F*_ST_ = 0.32), while the least differentiated wild populations were PG and SV (*F*_ST_ = 0.054) (S5 Table). The high pairwise population Fst values (S5 Table) observed between the AR, EV and MC wild populations indicate those populations were probably undergoing differentiation process, concurring with the data presented in Fig 2. The remaining wild populations presented lower pairwise *F*_ST_ values (S5 Table), indicating a lower genetic differentiation.

### Population structure

The 121 individuals of *C*. *cardunculus* were further evaluated for population stratification based on the admixture model approach using STRUCTURE software [46]. When SSR data were analysed, the number of subpopulations (K) tested were increased from one to 10. Estimation of ΔK by LnP(D) and Evanno’s ΔK method analysis, revealed the highest value for K = 2 (ΔK = 159.78), while K = 3 (ΔK = 3.17) and K = 8 (ΔK = 19.21) presented also the high levels of K (S2 Fig; S6 Table).

**Fig 3:**
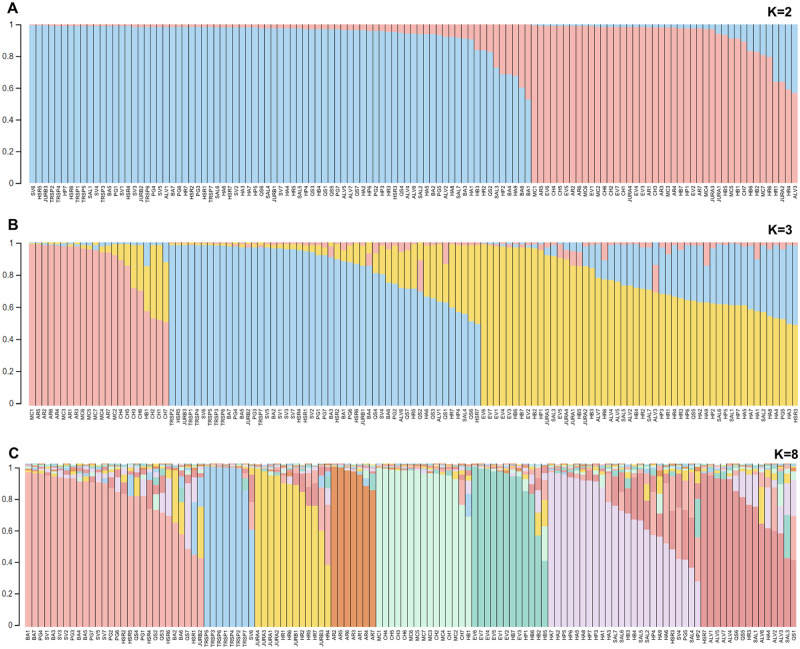
Structure analysis based on 121 genotypes of *Cynara cardunculus*. Genetic clusters inferred from the population structure analysis at K = 2 (A), 3 (B) and 8 (C), are represented by individual colors. Each sample (represented by a vertical bar) is partitioned into colored segments representing the estimated membership coefficients (*q* values). Genotypes names (geographic location followed by genotype number) are indicated on the x axis. Bar colors indicate the groups identified through the STRUCTURE program.

According to these results (K = 2) the geographic locations of CH, MC, AR, EV were, clustered together, included in group 2 (pink, Fig 3A). Most genotypes from these locations presented a very high membership coefficient, *q value*, above 0.80. Furthermore, some individuals from other geographic locations, HB (HB1, HB5 and HB7), JURA (JURA1, 3 and 4) and HP1, clustered in the same group 2 (q>0.80). However, genotypes HR1, JURA2, HR4 and ALV3 appears admixed, with a q value below 0.63. Other individuals from the BA, QS, PG, SAL, SV, HA, ALV, HP, HSR, JURB and TR populations were included in group 1 (blue, Fig 3A).

Based on the K = 3 model (Fig 3B), genotypes from geographic locations MC and AR, and CH4/CH5 genotypes from CH population were still clustered in the same group (pink), with a *q value* above 0.80. However, genotypes CH1, CH2 and CH7 showed a *q value* of approximately 0.5, indicating an admixed ancestry. In the blue group, EV appears jointed with JURA (JURA1-4), individuals from HB (HB2, HB3, HB5, HB6, HB7), HP1 and SAL3, with a high q value (>0.80). The second higher value of ΔK was observed at K = 8. Individuals from TR, AR and EV were maintained as distinct subgroups within the structure of the population (q>0.80), while CH (CH1 to CH6) and MC still joint in the same cluster (pink; Fig 3C). The CH7 genotype showed admixed with a q value of 0.75.

The differentiation of EV, CH, MC and AR locations was observed in PCoA analysis, and is also supported by Fst values and confirmed by populations STRUCTURE analysis. In addition, the admixture model approach shows there is a consistent structure within the Juromenha location, concerning JURA and JURB populations, which is indicated by the K = 2 and K = 3 analyses. Although individuals from JURA and JURB are located within the same geographic area, genotypes of JURA are in fact isolated from JURB by a water barrier.

**Fig 4:**
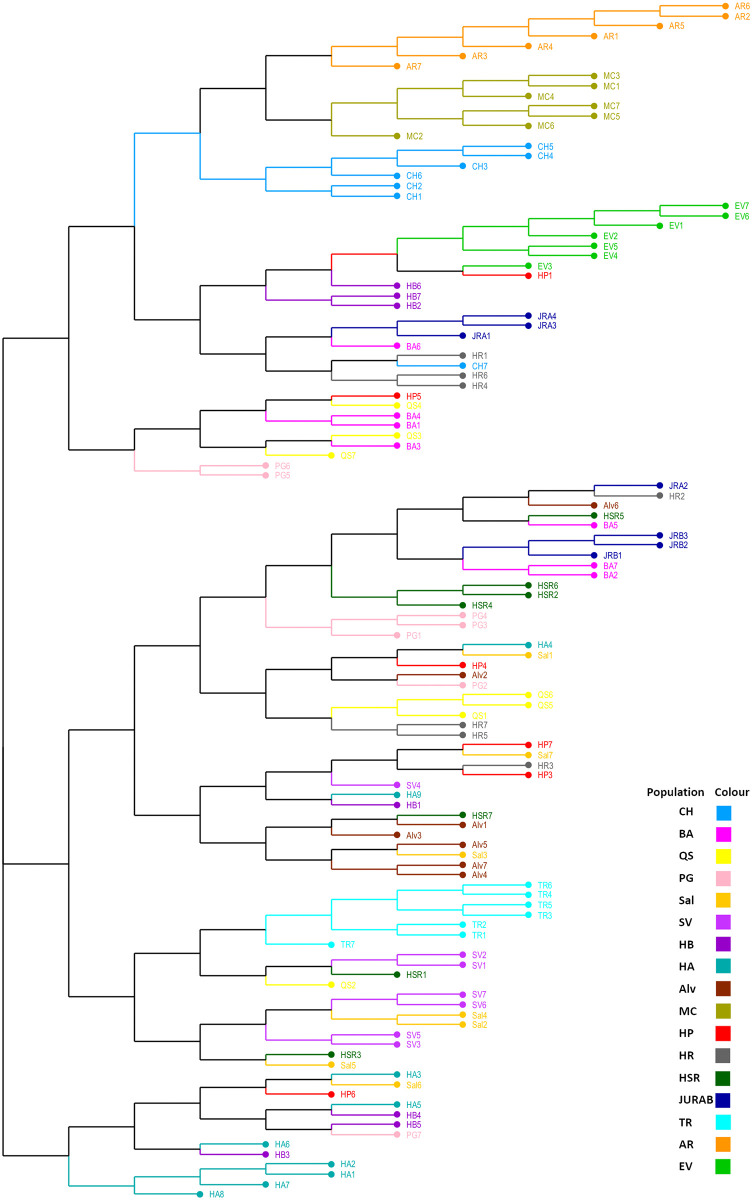
NJ phylogenetic tree showing affiliations of the *Cynara cardunculus* populations based on genetic dissimilarity of SSR microsatellite analyses. Geographic origin of populations is identified by a colour (see Table 1 for abbreviations of geographic populations).

A phylogenetic tree using neighbour-joining analysis, based on genetic distance, rendered three distinct groups (I, II, III) of *C*. *cardunculus* (Fig 4). This analysis, based on a dissimilarity matrix, grouped AR, MC, CH, EV and some individuals from HB, and JURA in cluster I. The second cluster grouped individuals from TR, SAL, SV and HSR among others. The HA population was assigned to an independent cluster, cluster III.

## Discussion

### Microsatellite polymorphism and genetic diversity of *Cynara cardunculus*

Characterization of genetic diversity is fundamental to design and manage strategies for species conservation and breeding programs. However, concerning Portuguese *C*. *cardunculus* germplasm there are only few studies supported by molecular markers, across its natural distribution [33, 35]. This study encompasses several wild genotypes of *C*. *cardunculus* from different geographic locations in Portugal. Our study is comprehensive, and thus an important contribution to improve the ability to assess and manage *C*. *cardunculus* germplasm in Portugal. Consequently, it provides a contribution to our understanding of this species germplasm characterization also in the Mediterranean region, supplementing the limited information currently available [35].

Efficiency of molecular markers, for population studies, is largely dependent upon their ability to detect levels of polymorphism. The mean number of alleles per locus observed in our study (N = 13.8) was higher than in prior studies of *C*. *cardunculus* [32, 34, 38, 50]. According to Botstein *et al*. [51] eight SSR loci were found to be highly informative (PIC > 0.5), with two others being moderately informative (0.5 > PIC > 0.25). The mean PIC of SSRs collectively used in our study was higher than those used in previous *C*. *cardunculus* genetic diversity studies using nuclear microsatellites [34, 38, 50]. Our findings indicate that the SSR markers we used were sufficiently informative to suitably evaluate Portuguese *C*. *cardunculus* germplasm.

In our study, relatively high allelic diversity and heterozygosity confirm the high level of diversity represented by *C*. *cardunculus* populations of southern Portugal found in the wild, perhaps related to the high level of outcrossing. The genetic diversity of Portuguese populations of cardoon in this study was higher (He = 0.591) than the wild cardoon populations from other Iberian populations (He = 0.370), studied by Gatto *et al*. [33], based on SSR markers. The lower genetic diversity observed by Gatto *et al*. [33] could be related to lower number of genotypes (n = 5) analyzed. Within the present work, the genotypes from the ALV, HA, HR and HSR populations presented the highest genetic diversity, compared to those from other geographic locations. The mean Ho detected in our study was lower than mean He, which might indicate population isolation. Similar lower levels of Ho were also detected in seeds from leafy cardoon and wild cardoon, indicating that natural cross-breeding occurs in populations in the wild, as also noted by other authors [32, 33]. However, some populations have similar values for He and Ho (fixation index F, close to zero) like PG, ALV indicating that the population are under Hardy-Weinberg Equilibrium (HWE), undergoing random mating, without significant natural selection, gene migration, mutation, or genetic drift [52]. The fixation index (F), also referred to as the Inbreeding Coefficient, represents any deviation from HWE, and allows detection of inbreeding, population fragmentation, migration and selection [53]. Most Portuguese populations showed more homozygous genotypes than expected (F positive), with exception of those from the CH, PG, HA, ALV, TR and AR locations. One factor explaining the relatively lower heterozygosity found in some geographic locations might be a result of inbreeding, resulting from small population size. The mean Fixation index from Portuguese germplasm in our study was 0.026, similar to that found for Iberian wild germplasm (F = 0.024), described by Gatto *et al*. [33]. In this study, wild populations from more easterly locations in Europe, such as those from Italy, Greece, Tunisia and Malta had a slightly higher level of inbreeding. Correspondingly, those populations were more homogeneous when compared to Iberian wild germplasm, from Portugal and Spain.

Level of genetic diversity of a species can be dependent on length of life cycle, reproduction system, geographic range and gene flow [53]. In our study, we found the majority of genetic variation occurred within populations of cardoon from the same geographic location. This finding is in accordance to previous studies of other cardoon populations described by others [33, 50, 54]. Higher diversity of *C*. *cardunculus* within the same geographic area might be related to the high level of outcrossing and degree of wind pollination inherent to cardoon. Such processes would increase gene flow between populations and reduce differentiation among them. For decades, cardoon has been used for multiple purposes having a wide range of applications, such as in traditional cheese production, human nutrition, and more recently for energy purposes [55, 56]. Hence, human factors may have also played a role in the higher level of genetic diversity detected within population of *C*. *cardunculus* in Portugal, while variation was lower among groups. Although the actual contribution of human activity to the rate of gene flow is unknown, low levels of differentiation among some geographical groups might reflect human activity in different regions contributing to exchange of germplasm. In addition, pollination of cardoon is chiefly performed by insects or mechanical (wind) agitation, according to Harwood and Markarian [57]. Cardoon has been considered as a species that predominantly out-crosses, having a low capacity for self-fertilization [34]. This association has resulted in a high level of within-species genetic diversity in cardoon.

### Population structure and molecular phylogeny

The structure of a population affects the degree of its genetic variation and pattern of distribution [58]. Our study included an analysis of population structure based on the sampled populations in order to identify any domestication events, heretofore unknown.

The likely number of different subpopulations we found, K = 2, was estimated using computer-based clustering analysis available in STRUCTURE [46]. That analysis identified the genotypes from AR, EV and MC geographic locations in the same cluster, pink group (Fig 3), corresponding to the northern edge of the Alentejo region. Geographic isolation among *C*. *cardunculus* populations of northern Alentejo may have resulted in minimal gene flow among them in surrounding areas. Moreover, in the same pink group, the CH population forms a cluster having a high proportion of similar alleles. As far as we know, genotypes from the CH location descended from Portuguese seeds of unknown origin, introduced along ago, into Alentejo, considering the specific biochemical properties of the cardoon flower for cheese production. In addition, the HR population partly represents a relictual experimental field population, introduced for studies on genetic diversity in the region several decades ago. The seeds for this study originated from different parts of Portugal, namely Beja, Quinta-do-Marquês in Oeiras and Torre Vã in Santiago do Cacém. These different Portuguese sources, might explain the similar ancestry proportion in the pink group, of 3 genotypes (HR1, HR4 and HR6) from HR population.

From the phylogenetic tree using neighbour-joining (NJ) cluster analysis it is also inferred a clear clustering of AR, MC, CH, EV geographic locations (Fig 4). The remaining populations were more complex, suggesting they were genetically admixed. The wild conditions of these populations and high level of outcrossing could explain the admixture of ancestry observed in several genotypes.

## Conclusions

Understanding genetic diversity and population structure of *C*. *cardunculus* is critical for efficient management of its genetic characteristics when designing suitable cultivars. According to our results, identification of microsatellites using SSR markers, proved to be a reliable method to assess *C*. *cardunculus* population genetics. Our study is a significant contribution to the knowledge of cardoon genetics and genotypes of wild populations in southern region of Portugal.

The high level of genetic variability within the wild cardoon populations studied, provides essential information for future germplasm conservation. Moreover, this study showed there is significant genetic differentiation in the gene pools among various cardoon groups, namely those from the northern edge of the Alentejo region. This differentiation provides a robust, independent source of genetic variability and is a valuable resource of genetic traits for breeders. Choosing optimal cardoon reproductive material, based on our genetic diversity findings will help to support the stability of *C*. *cardunculus*. Moreover, previous studies showing variability in natural product profile and cynaropicrin content, the major SL presented in *C*. *cardunculus* leaves [14–16], merge our findings reflecting the high level of genetic variability in populations of Portuguese cardoon.

A molecular database reflecting the variability of *C*. *cardunculus* genotypes, with identified morphological and biochemical profiles, will be useful to develop new biotech strategies used in future breeding programs. Such programs could significantly be designed to enhance content of bioactive compounds having pharmaceutical and/or nutraceutical applications. The knowledge here disclosed, greatly contributes to augment the economic value of cardoon at both regional and national levels.

## Supporting information

S1 Fig Example of electropherograms showing the different alleles at the *locus* CELMS-58, for five genotypes of cardoon (SAL4, QS4, PG4, BA7, AR6). The red peaks at 250 (nt) represent the standard size marker.
(TIF)

S2 Fig Figures showing the four steps of the Evanno method used for detecting ideal number of populations, K value. A. Mean L(K) ± SD after five runs for each K value. B. Rate of change of the likelihood distribution (mean ± SD) calculated as L’ (K) = L (K)-L (K-1). C. Absolute values of the second order rate of change of the likelihood distribution (mean ± SD) calculated according to the formula: |L” (K)| = |L’ (K+1)–L’(K)|. D. ΔK calculated as ΔK = m|L”(K)|/ s(L(K)]. The modal value of this distribution is the true K, or the uppermost level of structures, here designating three clusters.
(TIF)

S1 Table Nucleotide sequences of 23 primer pairs used for PCR amplification of microsatellites (SSRs) used in our study and characterization of those generated SSRs based on nucleotide repeats, number of repeats, linkage group and expected allele size, described on the literature. The multiplex loci combination and the respectively dyes used during our work, are also indicated.
(XLSX)

S2 Table The nucleotide sequences of amplicons obtained using 10 selected SSR primer pairs for large scale PCR amplification. (XLSX)

S3 Table Allele frequencies by population and total of the 10 SSR loci. (XLSX)

S4 Table Percentage of variation explained by the first 3 axes using Principal coordinate analysis. (XLSX)

S5 Table Pairwise population Fst values. (XLSX)

S6 Table Table showing the data output of the Evanno method. The asterisk mark shows the largest values in the Delta K column.
(XLSX)
