# Supplementary figures and images for: Genetic diversity and population structure of Cynara cardunculus L. in southern Portugal
Source: PLoS One. 2021 Jun 9;16(6):e0252792. doi: 10.1371/journal.pone.0252792 (PMC8189484; doi:10.1371/journal.pone.0252792)

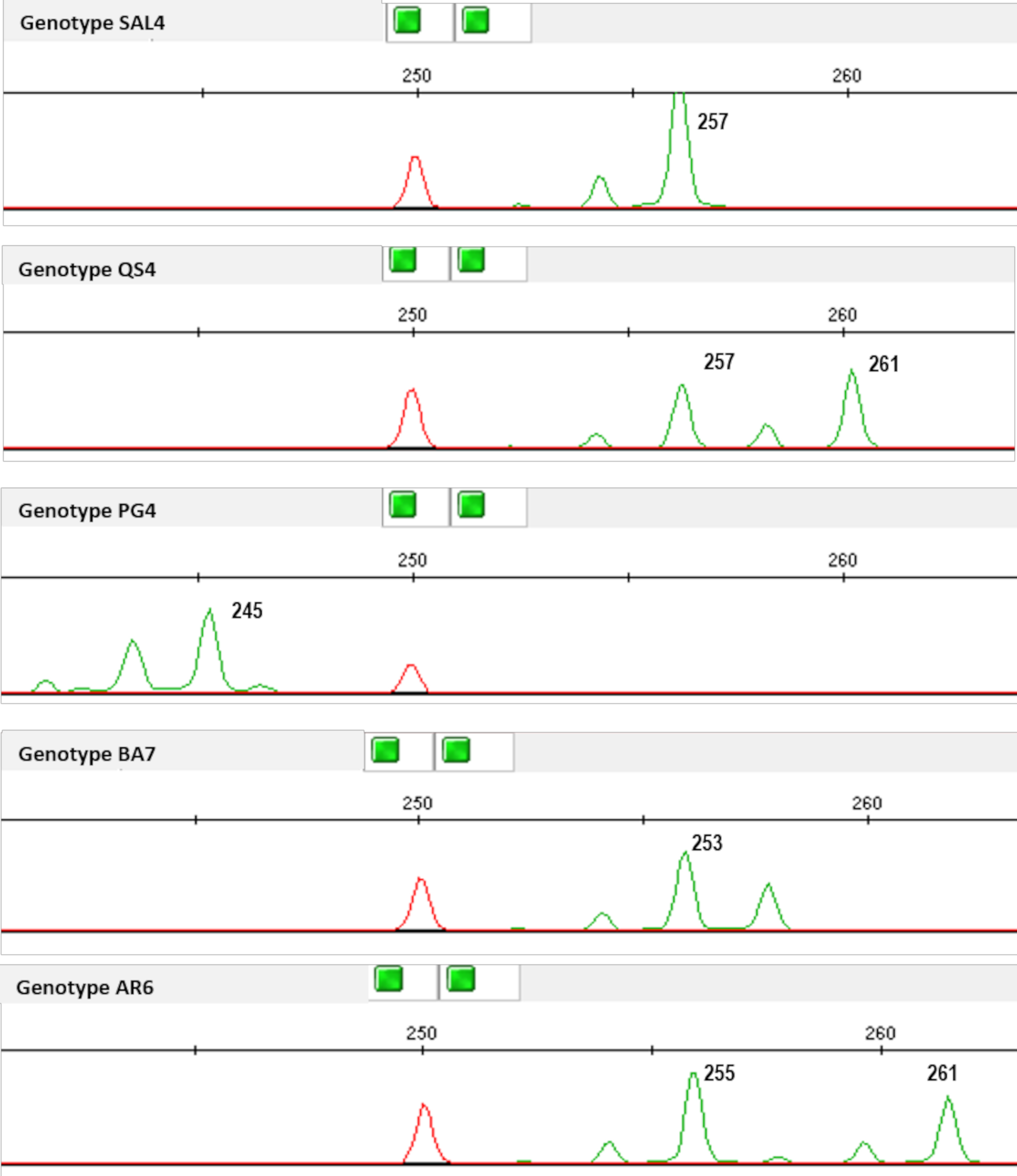

Supplement: S1 Fig — The red peaks at 250 (nt) represent the standard size marker. (TIF) [file pone.0252792.s001.tif]

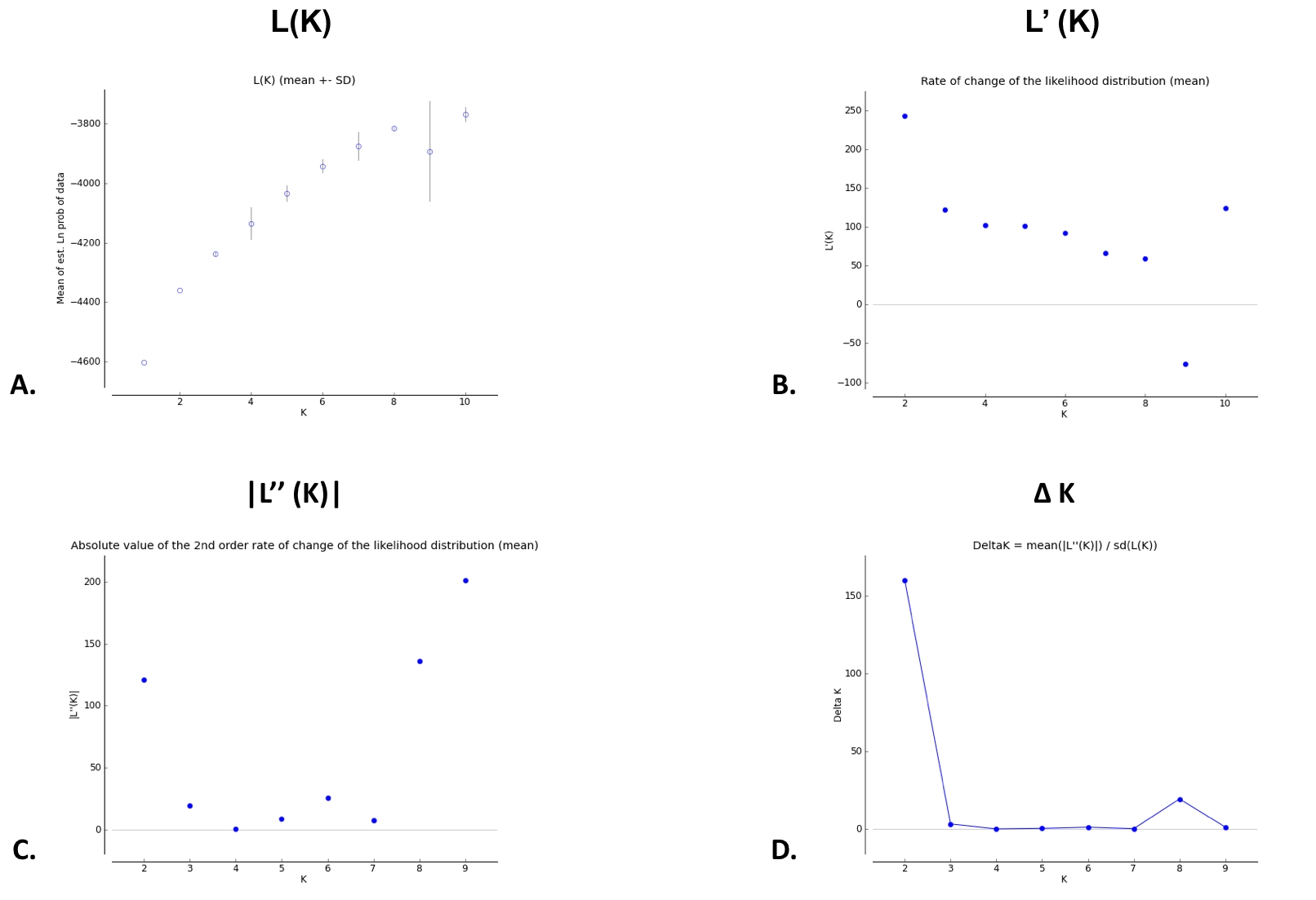

Supplement: S2 Fig — A. Mean L(K) ± SD after five runs for each K value. B. Rate of change of the likelihood distribution (mean ± SD) calculated as L’ (K) = L (K)-L (K-1). C. Absolute values of the second order rate of change of the likelihood distribution (mean ± SD) calculated according to the formula: |L” (K)| = |L’ (K+1)–L’(K)|. D. ΔK calculated as ΔK = m|L”(K)|/ s(L(K)]. The modal value of this distribution is the true K, or the uppermost level of structures, here designating three clusters. (TIF) [file pone.0252792.s002.tif]
